# A minor role of CD4^+ ^T lymphocytes in the control of a primary infection of cattle with *Mycoplasma mycoides *subsp. *mycoides*

**DOI:** 10.1186/1297-9716-42-77

**Published:** 2011-06-12

**Authors:** Flavio Sacchini, Jan Naessens, Elias Awino, Martin Heller, Andreas Hlinak, Wolfram Haider, Anja Sterner-Kock, Joerg Jores

**Affiliations:** 1 Istituto G. Caporale, Via Campo Boario, 64100 Teramo, Italy; 2 International Livestock Research Institute, Old Naivasha Road, PO Box 30709, 00100 Nairobi, Kenya; 3 Friedrich-Loeffler-Institut-Federal Research Institute for Animal Health, Naumburger Str. 96a, 07743 Jena, Germany; 4 Landeslabor Berlin-Brandenburg, Gerhard-Neumann-Str. 2-3, 15236 Frankfurt (Oder), Germany; 5 Institut für Tierpathologie, Schönhauser Strasse 62, 13127 Berlin, Germany; 6 Center for Experimental Medicine, University of Cologne, Medical School, University Hospital of Cologne, 50924 Cologne, Germany

## Abstract

Contagious bovine pleuropneumonia (CBPP), caused by *Mycoplasma mycoides *subsp. *mycoides*, is an important livestock disease in Africa. The current control measures rely on a vaccine with limited efficacy and occasional severe side effects. Knowledge of the protective arms of immunity involved in this disease will be beneficial for the development of an improved vaccine. In previous studies on cattle infected with *M. mycoides *subsp. *mycoides*, a correlation was detected between the levels of mycoplasma-specific IFN-γ-secreting CD4^+ ^T lymphocytes and reduced clinical signs. However, no cause and effect has been established, and the role of such cells and of protective responses acquired during a primary infection is not known.

We investigated the role of CD4^+ ^T lymphocytes in CBPP by comparing disease patterns and post mortem findings between CD4^+ ^T cell depleted and non-depleted cattle. The depletion was carried out using several injections of BoCD4 specific murine monoclonal antibody on day 6 after experimental endotracheal infection with the strain Afadé. All cattle were monitored clinically daily and sacrificed 28-30 days post-infection. Statistically significant but small differences were observed in the mortality rate between the depleted and non-depleted animals. However, no differences in clinical parameters (fever, signs of respiratory distress) and pathological lesions were observed, despite elimination of CD4^+ ^T cells for more than a week. The slightly higher mortality in the depleted group suggests a minor role of CD4^+ ^T cells in control of CBPP.

## Introduction

Contagious bovine pleuropneumonia (CBPP) is a livestock disease of high economic importance currently reported in many sub-Saharan African countries. Primary infection in cattle with *Mycoplasma mycoides *subsp. *mycoides *causes inflammation of the lung with respiratory symptoms and fever, that may progress into a lethal, generalized acute pleuropneumonia or lead to a chronic form with milder clinical signs and circumscribed pathomorphological lesions called sequestra. CBPP can be eradicated in countries having efficient veterinary services, and with the capacity to implement available disease control measures (test and slaughter policy, animal movement control and the provision of funds to compensate farmers). However, these measures are not applicable in most parts of Africa. The live T1/44 vaccine most commonly used in Africa induces immunity of short duration, which makes repeated vaccination campaigns necessary, and occasionally causes severe side effects. Knowledge of the nature of the protective response would greatly assist in the design of a better vaccine.

Although immunization with the live vaccine only confers immunity for up to one year [1], it means that immunological memory can be established. The exact nature of the protective response has not been determined. In the past, attempts were carried out to identify the mechanisms that trigger immunity towards *M. mycoides *subsp. *mycoides *infection. However, a critical review of past experiments does not provide clear evidence of the nature of protective immune responses in CBPP [2-4]. The existence of acquired immunity after vaccination led researchers to hypothesize that immune responses may be involved in protection during a primary infection and may contribute to a reduction in disease severity with subsequent development of a chronic form of disease. During primary infection, a correlation was reported between high numbers of mycoplasma-specific IFN-γ-secreting CD4^+ ^T lymphocyte subsets and a mild form of disease [5-8]. The data suggest that such cells, and thus acquired responses, might be involved in disease control. In another study no correlation was found between IFN-γ secretion of PBMCs and pathological outcome [9]. It is possible that differences with respect to the mycoplasma strain used for infection, the mode of infection and other environmental factors can alter the host immune responses and consequently protection. It is also possible that the number of animals used in the experiments was not high enough to make unambiguous conclusions, as pathological signs can vary considerably among individual animals. No study has ever demonstrated cause and effect.

To provide evidence for a protective role of IFN-γ secreting CD4^+ ^T cells, the total depletion of CD4^+ ^T cells should result in a dramatic increase in disease severity and mortality during a primary experimental infection. Even though the CD4^+ ^T cells consist of several regulatory subpopulations such as Treg and T helper cells we do expect a significant effect on disease control and pathology if a single subpopulation has a major role in disease control. Since a murine model of CBPP does not exist, the influence of CD4^+ ^T cells in CBPP was investigated in bovine infection. Almost complete elimination of peripheral T cell subpopulations in cattle has been achieved using large quantities of BoCD4 or BoCD8-specific murine monoclonal antibodies [10-12]. In this study, the role of CD4^+ ^T cells and acquired responses during a primary infection with *M. mycoides *subsp. *mycoides *was assessed by comparing clinical, pathological and laboratory parameters in CD4^+ ^T cell-depleted and control cattle.

## Materials and methods

### Animal experiment setup

All protocols of this study were designed and performed in strict accordance with the Kenyan legislation for animal experimentation and were approved by the institutional animal care and use committee (IACUC reference number 2008.08). Since 1993, ILRI has complied voluntarily with the United Kingdom's Animals (Scientific Procedures) Act 1986 that contains guidelines and codes of practice for the housing and care of animals used in scientific procedures.

Twenty castrated Boran cattle (*Bos indicus*), 14-16 months of age and randomly selected from the ILRI ranch in Kapiti (CBPP free-region in Kenya), were transferred to the ILRI campus in Nairobi and kept on pasture for 2.5 months. After arrival at the campus, all animals were dewormed twice using levamisole and treated prophylactically against babesiosis and anaplasmosis using imidocarb. The cattle were re-vaccinated against lumpy skin disease, anthrax & blackleg (Blanthax vaccine, Cooper) and twice against foot and mouth disease. All animals were tested for presence of antibodies against CBPP, East Coast Fever and trypanosomiasis using ELISA and found to be negative. One week before experimental infection, all animals were transferred to the animal biosafety level two (ABSL2) unit. All 20 cattle were infected on the same day. Cattle were sedated using 0.5 cc of 2% Xylazine solution/per animal and forced in lateral recumbency on the left side. A bronchial rubber tube was inserted deeply into the trachea and the infectious inoculum was introduced. Each animal received 50 mL of fresh *M. mycoides *subsp. *mycoides *Afadé liquid culture (2.5 × 10^10 ^colony forming units per animal) grown in pH medium [13], followed by 20 mL of a 1.5% heated agar solution and 30 mL phosphate buffered saline (PBS). The cattle were allowed to move freely within the SADF facility. Three veterinarians monitored the health status of the animals throughout the experiment. Rectal temperature was measured every morning. Blood samples for subsequent analysis were taken twice a week by jugular vein puncture. Animals showing fever for several consecutive days, abnormal behavior, breathing distress, or recumbency and inappetence were euthanized while the remaining animals were euthanized at 28-30 days post-infection (dpi).

CD4^+ ^T cells were depleted in 10 of 20 animals at day 6 post-infection (pi) (Table 1). CD4^+ ^T cell depletion was achieved by administering five consecutive intravenous injections of BoCD4 specific murine monoclonal antibody (ILA-11) [10] ascitic fluid starting with 50 μL followed by 200, 500, 1000 and 8650 μL at intervals of one hour. The concentration of the monoclonal antibody was 11.8 mg/mL.

**Table 1:** Summary of post mortem records of cattle after experimental infection with *Mycoplasma mycoides *subsp. *mycoides*

Animal **No**.	Lung side affected	Pulmonary marmorization	Pulmonary red hepatization	Pulmonary gray hepatization	Pulmonary necrosis	Pulmonary sequestra (size in mm)	Pulmonary pseudomembrane	Pulmonary fibrinous adhesion	Pleural fluid (liters)	Resolution of lesions	Remarks
**BD91 †**	Left			+++	+		++	++		++	
**BD93**	Left				Multifocal	10				++	
**BD94**	Left				Multifocal	20				++	Renal infarcts
**BD96**	Left				+					++	
**BD98 †**	Left	+	+		Multifocal	2-20				++	
**BD99**	Left				++	40				++	
**BD100**	Left				Multifocal	40				+++	
**BD101**	Left			++	++					++	
**BD118 †**	Left	++	+++	++	++		+++	++	3		Pericarditis
**BD119**	Left				+	10-60				++	
BD92	Left				Multifocal					++	
BD95	Left & right				+++					+++	
BD97 †	Right	+++	+++		+		+++	+++	7		Lung engorgement, many renal infarcts
BD102	No lesions										
BD105	Left				Multifocal	20-40				++	
BD106	Left									+	
BD107	Left			+++	Multifocal	20		+		++	Renal infarcts
BD111	Left				++	30-60		++		++	
BD115	Left				++	30				++	
BD116	Left				Multifocal					++	Renal infarcts

†-euthanized before 28 dpi, Animals displayed in bold have been depleted.

### Flow cytometry

Peripheral blood mononuclear cells (PBMCs) were harvested from blood using standard separation on Ficoll-Hypaque. Monoclonal antibodies GB21A, IL-A11 (IgG2a) or CC30 (IgG1), IL-A105, MM1A, IL-A24, [14] were used to detect γδ, CD4^+^, CD8^+ ^and CD3^+ ^T cells, and myeloid cells, respectively. Cells were stained indirectly [10], using a FITC-conjugated anti-mouse IgG or IgG1 or IgG2a (SouthernBiotech, USA). Flow cytometry was carried out on a FACSCANTO (Beckton Dickinson, Erembodegem, Belgium).

When testing peripheral blood for the presence of lymphocyte subpopulations post depletion, a control sample with FITC-conjugated second step antibody was added to monitor possible CD4^+ ^-antibody (IL-A11)-coated cells.

### Serology

Complement fixation test (CFT) (CIRAD, France) for CBPP was performed on all serum samples according to vendor's recommendations.

Additionally, sera were tested for antibodies to bovine herpes virus 1 (BHV-1), bovine respiratory syncytial virus (BRSV), bovine respiratory parainfluenza-3 (PI-3), bovine herpes virus 4 (BHV-4) and *Coxiella burnetii *using the HerdChek* IBRgB antibody test kit (IDEXX Europe BV, The Netherlands), Bio-X, BRSV ELISA Kit (Bio-X Diagnostics, Belgium), Bio-X Parainfluenza 3 ELISA Kit, (Bio-X Diagnostics, Belgium), Bio-X BHV-4 ELISA Kit (Bio-X Diagnostics, Belgium), and CHEKIT Q-Fieber antibody test kit (IDEXX Europe BV, The Netherlands), respectively according to vendors recommendations.

### Microbiology

Lung samples, carpal joint fluid, and pleural fluid specimens taken at necropsy were used for isolation of *M. mycoides *subsp. *mycoides *using modified pH medium as described before [13]. Lung samples and pleural fluid were used for screening of *Pasteurella *and *Mannheimia *spp. using standard methods [15].

### Statistical analysis

Exact and normal approximation binomial tests were used to compare the CD4+ T cell depleted group and the control group using GenStat 12th Edition [16]. P values for differences in fever and complement fixation test were estimated using a 2-sided 2-sample t-test comparing average levels between depleted and control animals at 5% level of significance.

## Results

### Depletion of CD4^+ ^T lymphocytes

**Figure 1:**
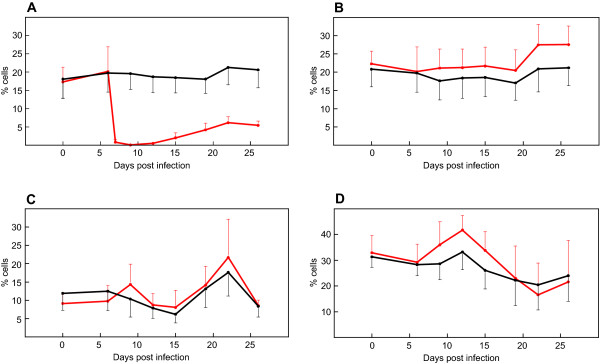
**Average percentage of peripheral lymphocyte subpopulations in cattle during experimental infection**. Lymphocyte subpopulations of CD4^+ ^(A), CD8^+ ^(B), γδ^+ ^(C), and B^+ ^(D) are displayed. Values were generated using FACS staining of PBMCs from ten CD4^+ ^T cell depleted (red) and ten control cattle (black) employing monoclonal antibodies specific for corresponding leucocyte markers. Standard deviations are displayed as bars.

The level of CD4^+ ^T cells in peripheral blood was recorded using flow cytometry throughout the experiment (Figure 1). Administration of monoclonal antibody ILA-11 resulted in a decrease in the number of peripheral blood CD4^+ ^T lymphocytes to below detectable levels. The number of CD4^+ ^cells measured by IL-A11 (IgG2a) or CC30 (IgG1), in combination with isotype-specific second step reagents, was always similar (data not shown). We did not detect CD4^+ ^antibody (IL-A11) coated cells the first two days after depletion (data not shown).

Leukocyte populations such as CD8, γδ T cells and myeloid cells (Figure 1) were also monitored and found not to be affected by the depletion experiment, except that their percentage of the total white blood cell counts increased due to the disappearance of CD4^+ ^T lymphocytes.

### Clinical observations

**Figure 2:**
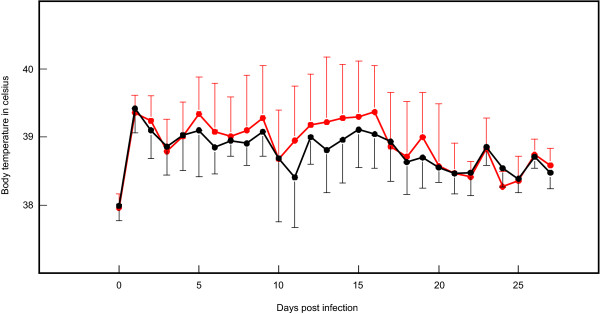
**Average body temperatures during experimental infection**. Values were generated using daily rectal temperatures from ten CD4^+ ^T cell depleted (red) and ten control cattle (black). Single standard deviations are displayed as bars.

Prior to experimental infection no study animals showed clinical signs of disease while being housed at ILRI in Nairobi. Immediately after experimental infection, all animals developed pyrexia (Figure 2), most likely resulting from inflammation of the respiratory system due to inoculum application. Several consecutive days of high fever (> 39.5°C) were observed in animals from both groups, about a week after experimental infection. Despite several peaks observed in a few animals, the average temperatures did not differ statistically (*p *= 0.377) between the groups (Figure 2).

**Figure 3:**
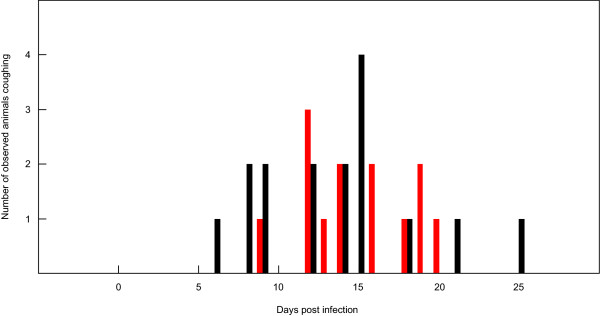
**Animals recorded coughing during experimental infection**. Values were generated using ten CD4^+ ^T cell depleted (red) and ten control cattle (black) observed daily for coughing.

All animals were observed for at least one hour per day to monitor clinical health status, including coughing. The highest number of animals coughing was observed between days 12-15 pi, corresponding with the clinical peak of acute disease in animals (Figure 3). Differences in breathing distress and the number of animals affected between the groups were not observed. Four animals were euthanized because of disease severity, three from the depleted group (BD91, BD98, and BD119) and one from the control group (BD97). The percentage of CD4^+ ^T cells in the latter four animals did not differ from those of the surviving animals in the same group.

### Pathological observations in infected cattle

Animal euthanasia was planned 28-30 dpi (22-24 days post depletion). One control and two depleted animals were euthanized on day 16 (BD97, BD91, and BD118) and another depleted animal (BD98) on day 21 pi. A 1-sample exact binomial test to evaluate if mortality in the depleted group was significantly higher than the control group showed evidence of an increase (*p *= 0.070).

Post mortem examination showed the presence of macroscopic lesions in 19 out of 20 animals, confirming successful experimental infection. The pathological findings are summarized in Table 1. Typology, extension and lesion severity were variable among the animals within the groups; no obvious differences between the two animal groups were observed. The control group had the only animal without any pathological lung lesions (BD102).

### Mycoplasma isolation and seroconversion

*M. mycoides *subsp. *mycoides *was isolated from lung or pleural fluid specimens from all infected animals. The presence of *M. mycoides *subsp. *mycoides *was confirmed using a pathogen specific polymerase chain reaction targeting the *lppQ *gene [17].

**Figure 4:**
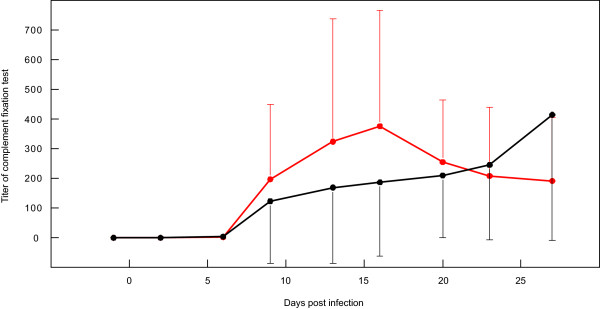
**Average CFT titers of CD4^+^-depleted and control animals during experimental infection**. Single standard deviations are displayed as bars.

Seroconversion was seen in all animals as shown by CFT titers (Additional file 1). The average titers measured between day 13 and day 27 pi did not differ statistically (*p *= 0.637) between the groups (Figure 4).

### Diagnosis of pathogens other than *M. mycoides *subsp. *mycoides*

We did not isolate *Pasteurella multocida *or *Mannheimia haemolytica *from the lung specimens taken at necropsy using standard methods. Serological analysis conducted to investigate the serum response to selected bovine pathogens (*Coxiella burnetii*, BRSV, BHV-1, BHV-4, PI-3) showed a striking increase in antibody responses against BHV-4 after experimental infection in both groups (Table 2).

**Table 2:** Antibody responses of cattle to BHV-1, BHV-4, BRSV, PI-3, and *Coxiella burnetii *before and after experimental infection with *Mycoplasma mycoides *subsp. *mycoides*.

Animal **No**.	BHV-1 (pre infection)	BHV-1 (post infection)	BRSV (pre infection)	BRSV (post infection)	PI-3 (pre infection)	PI-3 (post infection)	BHV-4 (pre infection)	BHV-4 (post infection)	*Coxiella burnetii* (pre infection)	*Coxiella burnetii *(post infection)
**BD91 †**	-	-	+	-	+++			++++		
**BD93**	-	-	-	-	++			+		
**BD94**	-	-	+	-				++++	+	+/-
**BD96**	-	-	+	-	+			+		
**BD98 †**	-	-	-	+	+	+				+/-
**BD99**	-	-	+	-						
**BD100**	-	-	-	-	+					
**BD101**	+	+	+	-						
**BD118 †**	-	-	-	-				++		
**BD119**	-	-	-	-	+					
BD92	-	-	+	+	++	+				
BD95	-	-	+	++	+	+		+		
BD97 †	-	-	-	+	+			+		
BD102	-	-	-	-		+				
BD105	+/-	-	-	-	++	++		++		
BD106	-	-	-	-	+	+		+		
BD107	-	-	-	-						
BD111	-	-	-	-	+	+				
BD115	-	-	+	+				+		
BD116	-	-	-	+	+	+		+		

BHV-Bovine Herpes Virus; BRSV-Bovine Respiratory Syncytial Virus, PI-3-Parainfluenza 3, †-euthanized before 28 dpi, Animals displayed in bold have been depleted.

## Discussion

Conflicting opinions for the role of CD4^+ ^T lymphocytes in CBPP have been published [5,9]. These are based on the presence or absence of a correlation between high numbers of IFN-γ-secreting CD4^+ ^T cells with mild disease outcome. As these studies never tested cause and effect, the aim of this study was to whether *M. mycoides *subsp. *mycoides*-specific CD4^+ ^T lymphocytes can alter the course of primary CBPP infection. We compared two groups of 10 animals, each infected with *M. mycoides *subsp. *mycoides *strain Afadé, one depleted for CD4^+ ^T lymphocytes six days pi and the other left untreated. Clinical parameters such as fever and cough, as well as post mortem results were recorded. Ten animals per experimental group were chosen based on statistical considerations and on the assumption that the depleted group would show a dramatic increase in disease severity if the CD4^+ ^T cells were involved in control of the infection.

A total of 118 mg/animal of the CD4^+^-specific monoclonal antibody IL-A11 was administered intravenously in five increasing doses, to achieve in vivo depletion of CD4^+ ^T cells. Depletion was successful as confirmed by flow cytometry showing no CD4^+ ^cells one day after treatment in peripheral blood. From previous depletion experiments, it was assumed that CD4^+ ^T cells were also completely removed from lymphoid tissues, with exception of the thymus [10]. Naïve CD4^+ ^cells could be detected in low numbers about one week later. The CD4^+ ^T cell numbers remained below 25% of normal throughout the entire experiment. The data shows that removal of CD4^+ ^T cells did not result in clear differences between both groups. No significant differences were found in fever or the frequency of coughing, both good correlates of clinical CBPP severity. Additionally, the number and size of lung lesions did not differ between both groups. The difference observed in mortality (3/10 versus 1/10), or more correctly in the number of animals with symptoms that necessitated euthanasia, was statistically significant (*p *< 0.07), though low and could be interpreted as being caused by loss of a protective response. The numbers demonstrate that the effect of depletion on mortality is minor.

In order to ensure that the depletion of a T cell population did not increase the presence of unrelated pathogens that could confound the pathology, the presence of a number of possible bacteria and viruses were monitored before and after infection. In related mycoplasma, such as *M. bovis *[18] and *M. hyopneumoniae *[19], co-infections with other pathogens which may contribute to lung pathology have been observed. Evidence for the presence of several viruses was detected in our study, however, no major differences were observed between the two groups. Interestingly we observed a substantial increase in antibody against BHV-4 after infection in both groups, which suggests an immuno-compromised state in the animals during clinical CBPP. We speculate that additional pathogens may affect CBPP infected animals, which could result in even more acute clinical symptoms.

One concern is that low numbers of CD4^+ ^T cells can mediate a protective function. This is difficult to accept as low numbers of IFN-γ-secreting CD4^+ ^T cells in acute animals were not sufficient to prevent acute disease [7]. Another concern is that the role of CD4^+ ^T cells is compensated by another cell type in treated animals. This cannot be disproven, but is unlikely. The CD4^+ ^population contains cells that provide regulatory functions, such as helper and suppressor tasks, which other cell types cannot do. Also, it is unlikely that depletion of peripheral IFN-γ-secreting CD4^+ ^T cells can trigger a compensating mechanism, while very low numbers (in acutely infected cattle) cannot. Furthermore, no compensation in number of other populations was observed in previous CD4^+ ^depletion experiments [20].

A reduction in antibodies in the depleted animals measured by the complement fixation test was not observed, as would be expected after eliminating a large portion of T cell help. A reduction in antibody production has been observed in previous CD4^+ ^depletion experiments in cattle or sheep [20-22]. It is possible that antibody production had begun prior to the depletion treatment. This seems unlikely as antibodies were first detected in blood by day nine and the kinetics of the antibody responses after that time did not statistically differ between treated and control groups. Another explanation is that anti-mycoplasma antibodies might be produced without T cell help. Such T cell-independent antibodies have been described in trypanosome infections, where antibody titres in infected *nu/nu *mice were only slightly lower than in normal mice [23]. Mycoplasma express high amounts of carbohydrate on their surface [24], which might form repetitive epitopes that need less T cell help to induce specific antibodies.

One final concern is the timing of depletion. We depleted six dpi, since previous experimental infections carried out in Kenya and Namibia suggested that the peak of clinical symptoms occurred around 2-3 weeks pi [9,25] and previous depletion experiments suggested complete elimination of T cells for one to two weeks after treatment. We succeeded in removing the CD4^+ ^T cells before the peak of clinical symptoms, but one can argue that a protective CD4 response acted prior to the depletion, or after CD4^+ ^T cells returned. If the protective immune response in animals with mild symptoms of disease was driven by high numbers of IFN-γ-secreting CD4^+ ^T cells, we would not expect such cells to appear early, as demonstrated in the previous studies [7,8]. Also, while naïve CD4^+ ^T cells appeared from the thymus one week after treatment, one would expect that a hypothetical, protective CD4^+ ^T cell response would be seriously delayed in the depleted animals, leading to severe disease.

If CD4^+ ^T cells do not provide the protective response in a primary infection, what defines whether an animal develops severe (acute) or a less severe (chronic) diseaseż Since there is no direct correlation between antibody titres and disease severity, it is difficult to ascribe a protective role to high antibody titers. Therefore, we assume that innate responses may play an important role. Of course, our data do not rule out a protective role for CD4^+ ^T cells in combination with antibody production after vaccination or during a secondary infection.

CBPP is a fatal disease particularly in naïve herds where up to > 30% of animals may die [26], indicating that during a primary infection the induced responses are not sufficient or appear too late to adequately control the disease. Following this reasoning, it is not surprising that acquired responses driven by CD4^+ ^T cells only play a minor role in control of primary CBPP infection as previously speculated [9]. In our opinion future research efforts towards the identification of protective immune responses should be focused on an infection model employing immune animals.

## Competing interests

The authors declare that they have no competing interests.

## Authors' contributions

JJ designed the study and drafted the manuscript with the help of JN. FS and JJ did the infection, depletion and monitoring of the animals throughout the entire experiment and were assisted by JN. EA did the FACS analysis. MH did the CFT analysis. AH did the serological analysis of BHV-1, BHV-4, BRSV, PI-3, and *Coxiella burnetii*. ASK and WH did to the post mortem analysis and were assisted by FS, JN, JJ, and MH. JJ coordinated the whole study. All authors read and approved the final manuscript.

## Supplementary Material

Additional file 1 **Table S1**: Individual serological responses of infected cattle against *Mycoplasma mycoides *subsp. *mycoides *measured by complement fixation test. Click here for file
