# Supplementary material for: A minor role of CD4+ T lymphocytes in the control of a primary infection of cattle with Mycoplasma mycoides subsp. mycoides
Source: Vet Res. 2011 Jun 12;42(1):77. doi: 10.1186/1297-9716-42-77 (PMC3148206; doi:10.1186/1297-9716-42-77)
Supplement: Additional file 1 — Table S1: Individual serological responses of infected cattle against Mycoplasma mycoides subsp. mycoides measured by complement fixation test. [file 1297-9716-42-77-S1.PDF]

Animal no. (day of euthanasia post infection)		Days post infection								
		-1	2	6	9	13	16	20	23	27
$CD4^+$ T CELL DEPLETED GROUP	BD91 (†16)	0	0	0	640	640	640			
	BD93 (†28)	0	0	0	80	80	640	320	80	80
	BD94 (†28)	0	0	0	10	80	160	320	320	160
	BD96 (†30)	0	0	0	80	40	40	20	20	20
	BD98 (†20)	0	0	0	40	320	320	640		
	BD99 (†30)	0	0	0	320	1280	1280	320	640	320
	BD100 (†29)	0	0	0	0	0	20	80	40	40
	BD101 (†29)	0	0	0	0	0	20	20	40	80
	BD118 (†16)	0	0	5	640	640	320			
	BD119 (†29)	0	0	10	160	160	320	320	320	640
CONTROL GROUP	BD92 (†29)	0	0	20	160	160	160	320	320	640
	BD95 (†30)	0	0	0	0	0	0	160	160	640
	BD97 (†16)	0	0	0	320	640	640			
	BD102 (†30)	0	0	0	10	10	10	10	10	10
	BD105 (†29)	0	0	0	0	0	0	0	0	80
	BD106 (†28)	0	0	0	0	0	20	40	40	40
	BD107 (†30)	0	0	0	0	0	80	80	80	80
	BD111 (†28)	0	0	0	640	640	640	640	640	640
	BD115 (†28)	0	0	10	80	160	160	320	320	320
	BD116 (†29)	0	0	5	20	80	160	320	640	1280
